# Elucidation of the Mechanism Underlying the Anti-Inflammatory Properties of (S)-(+)-Carvone Identifies a Novel Class of Sirtuin-1 Activators in a Murine Macrophage Cell Line

**DOI:** 10.3390/biomedicines9070777

**Published:** 2021-07-04

**Authors:** Cátia Sousa, Bruno Miguel Neves, Alcino Jorge Leitão, Alexandrina Ferreira Mendes

**Affiliations:** 1 Centre for Neuroscience and Cell Biology, University of Coimbra, 3004–504 Coimbra, Portugal; uc45185@uc.pt (C.S.); ajleitao@ff.uc.pt (A.J.L.); 2 Faculty of Pharmacy, University of Coimbra, 3000-548 Coimbra, Portugal; 3 Centre for Innovative Biomedicine and Biotechnology, University of Coimbra, 3004-504 Coimbra, Portugal; 4 Department of Medical Sciences and Institute of Biomedicine—iBiMED, University of Aveiro, 3810-193 Aveiro, Portugal; bruno.neves@ua.pt

**Keywords:** aging, inflammation, monoterpene, NF-κB, Sirtuin-1, Sirtuin-1 activating compound

## Abstract

The signaling pathways involved in age-related inflammation are increasingly recognized as targets for the development of preventive and therapeutic strategies. Our previous study elucidated the structure–activity relationship of monoterpene compounds derived from *p*-menthane as potential anti-inflammatory drugs and identified (S)-(+)-carvone as the most potent among the compounds tested. This study aims at identifying the molecular mechanism underlying the anti-inflammatory properties of (S)-(+)-carvone. The murine macrophage cell line, Raw 264.7, was stimulated with bacterial lipopolysaccharide (LPS) to simulate inflammation. Western blot was used to assess protein levels and post-translational modifications. The subcellular localization of NF-κB/p65 was visualized by immunocytochemistry. An in vitro fluorometric assay was used to measure Sirtuin-1 (SIRT1) activity. (S)-(+)-carvone inhibited LPS-induced JNK1 phosphorylation, but not that of p38 and ERK1/2 and also did not affect the phosphorylation and degradation of the NF-κB inhibitor, IκB-α. Accordingly, (S)-(+)-carvone did not affect LPS-induced phosphorylation of NF-κB/p65 on Ser536 and its nuclear translocation, but it significantly decreased LPS-induced IκB-α resynthesis, a NF-κB-dependent process, and NF-κB/p65 acetylation on lysine (Lys) 310. Deacetylation of that Lys residue is dependent on the activity of SIRT1, which was found to be increased by (S)-(+)-carvone, while its protein levels were unaffected. Taken together, these results show that (S)-(+)-carvone is a new SIRT1 activator with the potential to counteract the chronic low-grade inflammation characteristic of age-related diseases.

## 1. Introduction

Persistent low-grade inflammation represents a pathological mechanism associated with age-related diseases, such as metabolic, cardiovascular, neurodegenerative and musculoskeletal diseases and cancer [1]. A large range of stimuli, including inflammatory cytokines (e.g., Interleukin (IL)-1β, Tumor Necrosis Factor-α (TNF-α) and IL6), microbial products, cellular components released by dead or damaged cells (e.g., ATP and the alarmins, HMGB1 and members of the S100 family) [2] and intermittent hypoxia, especially in older people [3], activate multiple intracellular signaling cascades that bring about the inflammatory response. Of those signaling cascades, members of the Mitogen-Activated Protein Kinase (MAPK) family and the transcription factor, Nuclear Factor kappa-light-chain-enhancer of activated B cells (NF-κB), are especially relevant. Numerous studies have shown that their activation leads to the production of inflammatory mediators and effector enzymes that drive and perpetuate inflammation-associated tissue damage and functional impairment, thus promoting disease development and/or progression [2,4].

Considering the role of MAPKs and NF-κB in chronic inflammation and the lack of efficient therapeutic strategies for chronic inflammation-associated diseases, the signaling pathways that lead to their activation are promising targets for drug development [5,6].

**Figure 1:**
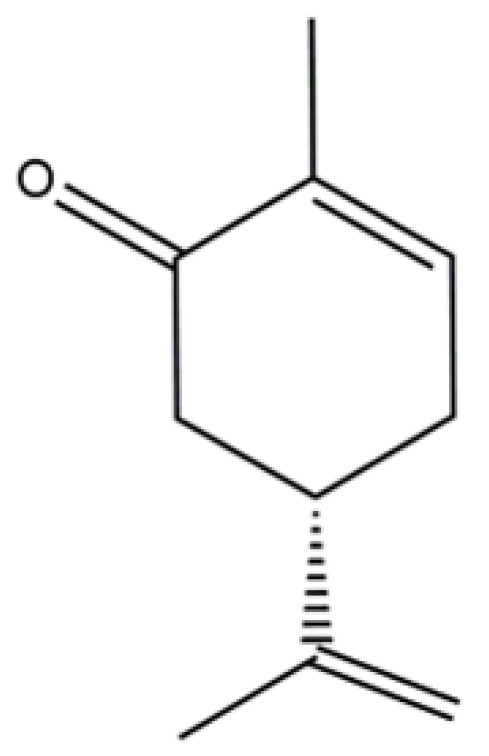
Structural formula of (S)-(+)-carvone.

Our previous work screened various compounds of natural origin in standardized conditions, to identify small molecules capable of interfering with those inflammatory pathways and to establish the structural features required for activity. (S)-(+)-carvone (Figure 1), a limonene-derived monoterpene especially abundant in mint species, was identified as the lead compound of that series, decreasing inducible Nitric Oxide (NO) Synthase (NOS2) and IL-1β expression, both in a mouse macrophage cell line and in primary human chondrocytes, in response to LPS and IL-1β, respectively [7]. These results are in line with other studies reporting anti-inflammatory [8], antioxidant [9], anti-hiperglycemic and hyperlipidemic [10,11] properties of (S)-(+)-carvone or the racemic mixture that also contains its (R)-(-) enantiomer. Nonetheless, the molecular mechanism(s) underlying those effects of (S)-(+)-carvone are not fully understood.

Therefore, the purpose of this work was to elucidate the molecular mechanism(s) by which (S)-(+)-carvone interferes with the expression of pro-inflammatory mediators. Considering the crucial role of MAPKs and NF-κB activation on pro-inflammatory gene expression, we hypothesized that these signaling pathways may be targeted by (S)-(+)-carvone. The results obtained confirm this hypothesis and allow for further insight into the molecular mechanism of action of (S)-(+)-carvone, showing that it directly activates Sirtuin-1 (SIRT1), a NAD+-dependent deacetylase known to target the p65 component of NF-κB, decreasing its transcriptional activity [12].

## 2. Materials and Methods

### 2.1. Cell Culture and Treatments

The mouse macrophage cell line, Raw 264.7 (ATCC No. TIB-71, Manassas, VA, USA), was cultured in DMEM (ThermoFisher Scientific, Walthman, MA, USA) supplemented with 10% non-inactivated fetal bovine serum (FBS; ThermoFisher Scientific), 100 U/mL penicillin (Sigma-Aldrich Co., St Louis, MO, USA) and 100 µg/mL streptomycin (Sigma-Aldrich Co.). Raw 264.7 cells were plated at a density of 3 × 10^5^ cells/mL and left to stabilize for up to 24 h. The cells were used between passages 25 and 40, as we verified that the usual responses to LPS are maintained in this range.

For cell treatments, (S)-(+)-carvone (#435759, purity 96%, Sigma-Aldrich Co.), resveratrol (Res; Extrasynthese, Genay Cedex, France), Bay 11-7082 (Calbiochem, San Diego, CA, USA) and MG-132 (Z-Leu-Leu-Leu-CHO, Boston Biochem, Cambridge, MA, USA) were dissolved in dimethyl sulfoxide (DMSO; Sigma-Aldrich Co.). LPS from Escherichia coli 026:B6 (Sigma-Aldrich Co.) was dissolved in phosphate-buffered saline (PBS). The concentrations of each compound and the experimental treatment periods are indicated in figures and/or figure legends. Concentrations of (S)-(+)-carvone were selected based on our previous work [6]. DMSO was used as vehicle and added to control and LPS-treated cell cultures to match the same concentration as in cells treated with the chemicals indicated above. In any case, the final concentration of DMSO was 0.1% (*v*/*v*). The chemicals used or the vehicle were added to murine macrophage cultures 1 h before the pro-inflammatory stimulus, 1 µg/mL LPS, and maintained for the rest of the experimental period.

### 2.2. Preparation of Cell Extracts

For the preparation of total cell extracts, cell cultures were washed with ice-cold PBS and lysed with ice-cold RIPA buffer [150 mM sodium chloride (ThermoFisher Scientific), 50 mM Tris (ThermoFisher Scientific, pH 7.5), 5 mM ethylene glycol-bis(2-aminoethylether)-N,N,N′,N′-tetraacetic acid (EGTA; Sigma-Aldrich Co.), 0.5% sodium deoxycholate (Sigma-Aldrich Co.), 0.1% sodium dodecyl sulfate (SDS; Sigma-Aldrich Co.), 1% Triton X-100 (Merck Millipore Ltd., Darmstadt, Germany) ] supplemented with protease (Complete, Mini, Roche Diagnostics, Mannheim, Germany) and phosphatase (PhosSTOP, Roche Diagnostics, Mannheim, Germany) inhibitor cocktails, for 30 min. The lysates were centrifuged at 14,000 rpm for 10 min at 4 °C and the supernatants were stored at −20 °C until use.

For the preparation of cytoplasmic and nuclear extracts, the Nuclear Extract Kit (Active Motif, La Hulpe, Belgium) was used, following the manufacturer’s instructions.

Protein concentration in the extracts was determined with the bicinchoninic acid kit (Sigma-Aldrich Co.).

### 2.3. Western Blotting

Western blot was performed as described previously [13]. Briefly, total (25 µg), cytoplasmic (25 µg) or nuclear (30 µg) proteins were separated by SDS-PAGE under reducing conditions. A commercial mixture of 12 purified pre-stained proteins (NZYColour Protein Marker II, NZYTech, Lisbon, Portugal) was run in each gel to allow for confirmation of the apparent molecular weight of the proteins of interest. The proteins were then electrotransferred onto PVDF membranes (Immobilon^®^—P, Merck Millipore Ltd.) which were probed overnight at 4 °C or for 2 h at room temperature with the primary antibodies indicated in Table 1 and then with anti-rabbit (dilution 1:20,000; NIF1317, lot9465473, GE Healthcare, Chalfont St. Giles, UK) or anti-mouse (dilution 1:20,000; NIF1316, lot6963606, GE Healthcare, Chalfont St. Giles, UK) alkaline phosphatase-conjugated secondary antibodies. Mouse monoclonal anti-β-Tubulin I and rabbit polyclonal anti-Lamin B1 were used as a loading controls of total and cytoplasmic extracts and of nuclear extracts, respectively. Immune complexes were detected with Enhanced ChemiFluorescence reagent (GE Healthcare) in the imaging system Thyphoon^TM^ FLA 9000 (GE Healthcare). Image analysis was performed with TotalLab TL120 software (Nonlinear Dynamics Ltd., Newcastle upon Tyne, UK).

### 2.4. Immunocytochemistry

Macrophages were seeded onto µ-Slide 8 Well chamber plates (ibiTreat, Ibidi, Martinsried, Germany) suitable for cell culture and microscopy, followed by immunostaining. After treatment, the cells were washed with ice-cold PBS pH = 7.4 and then fixed in 4% paraformaldehyde (Sigma-Aldrich Co.) at room temperature, for 15 min. After fixing, cells were washed three times with PBS pH = 7.4 with 0.1 M glycine (ThermoFisher Scientific) for 5 min each and blocked with 5% Goat Serum (Sigma-Aldrich Co.), 0.3% Triton X-100 in PBS, pH = 7.4 for 1 h at room temperature. Then, the slides were incubated with a rabbit monoclonal anti-NF-κB p65 (D14E12) XP^®^ antibody (dilution 1:400; #8242, Lot 4, Cell Signaling Technology, Inc., Danvers, MA, USA) in 1% Bovine Serum Albumin (Merck Millipore Ltd.) in PBS (pH = 7.4), overnight at 4 °C. The cells were washed three times with PBS (pH = 7.4) for 5 min each at room temperature and incubated for 1 h at room temperature with anti-rabbit IgG (H + L) CF^™^488A antibody (dilution 1:400; SAB4600165, Lot 10C0615 Biothium, Inc., Fremont, CA, USA). Following three washes with PBS (pH = 7.4), the cells were counterstained with DAPI (0.2 ng/mL; Molecular Probes, Invitrogen, Eugene, OR, USA) to allow for nucleus visualization, and after another washing step, the slides were mounted with Ibidi Mounting Medium (Ibidi, Martinsried, Germany). Specificity was evaluated in negative controls set up by omitting the primary antibody. Fluorescence images were obtained in an Axio Observer ZI fluorescence microscope (Carl Zeiss, Germany).

### 2.5. SIRT1 Activity Assay

Interaction of (S)-(+)-carvone with human SIRT1 was evaluated using the SIRT1 Direct Fluorescent Screening Assay Kit (Cayman Chemical Company, Ann Arbor, MI, USA) following the manufacturer’s instructions. Briefly, the assay uses a specific substrate, in this case, a peptide derived from the p53 sequence, coupled to a fluorophore (Arg-His-Lys-Lys(e-acetyl)-AMC), which is incubated with recombinant human SIRT1 along with its co-substrate, NAD^+^. Deacetylation sensitizes the substrate such that treatment with a developer reagent releases a fluorescent product. The activity of the enzyme is proportional to the fluorescence intensity. The ability of resveratrol to activate the enzyme was evaluated in parallel assays as a positive control. The results are presented as mean fluorescence intensity (arbitrary units) ± SEM.

### 2.6. Statistical Analysis

The results are presented as mean ± SEM. Statistical analysis was performed using GraphPad Prism version 6.0 (GraphPad Software, San Diego, CA, USA). Statistical significance was evaluated with the t-test to compare each condition with its respective control or with one-way ANOVA with the Dunnett post-test for comparison of multiple conditions to a control group. A non-parametric test (Mann–Whitney test to compare each condition to the basal SIRT1 activity) was used to assess the statistical significance of the differences observed in Figure 7c, as those results did not follow a normal distribution. The results were considered statistically significant at *p* < 0.05.

## 3. Results

### 3.1. (S)-(+)-Carvone Decreases JNK1 Phosphorylation Induced by LPS in Macrophages

The effects of (S)-(+)-carvone on activation of the three MAPK subfamilies, namely, Extracellular-signal Regulated Kinase (ERK) 1/2, p38 and Jun N-terminal Kinase (JNK), were evaluated by measuring their phosphorylated levels in response to macrophage stimulation with LPS.

**Figure 2:**
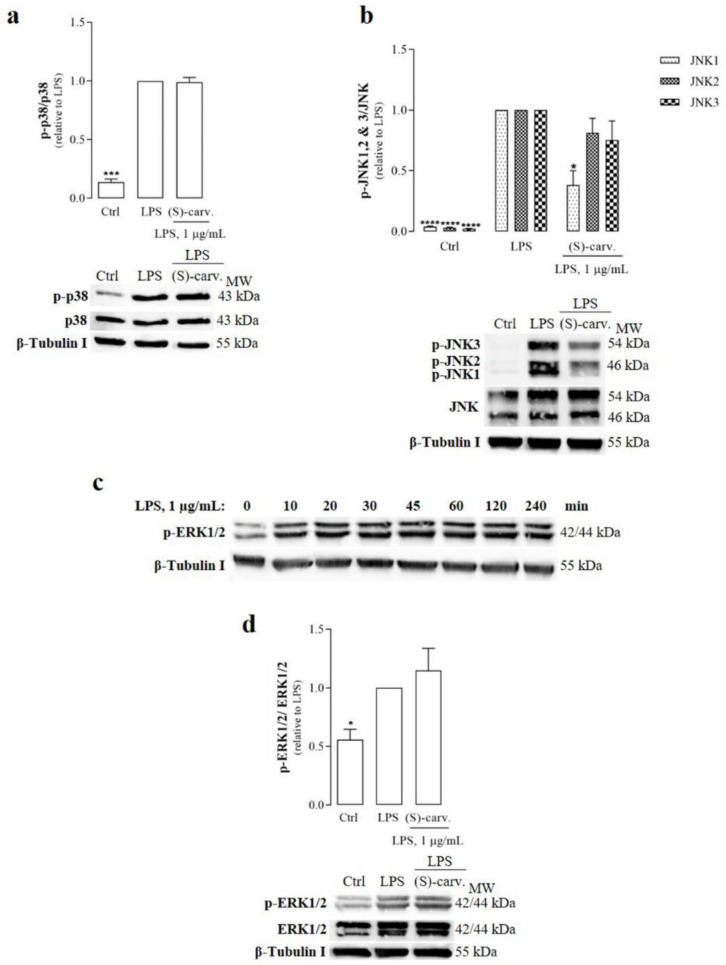
Effect of (S)-(+)-carvone on p38 (**a**), JNK (**b**) and ERK1/2 (**d**) activation. Raw 264.7 macrophage cultures were pre-treated with 665 µM (S)-(+)-carvone [(S)-carv.] for 1 h before addition of 1 µg/mL LPS for 5 min (**a**) and (**b**), the time periods indicated in (**c**) or 1 h (**d**). Control cells (Ctrl) were treated with the vehicle alone (0.1% DMSO) for the same time periods, except in **c**, where control cells were untreated. Each column represents the mean ± SEM of four (**a**) and (**b**) or three (**d**) independent experiments. Representative images are shown. * *p* < 0.05, *** *p* < 0.001 and **** *p* < 0.0001 relative to LPS-treated cells. MW: molecular weight marker.

The results obtained show that LPS induced the phosphorylation of p38 (Figure 2a) and JNK (Figure 2b) very rapidly, being detectable as early as five minutes after the addition of LPS. Pre-treatment with (S)-(+)-carvone had no effect on p38 phosphorylation (Figure 2a), while significantly decreasing JNK1 phosphorylation to, approximately, 38% of the levels found in cells treated with LPS alone (Figure 2b). Furthermore, a tendency for reduced JNK2 and 3 phosphorylation was also observed, but in no case did it reach statistical significance (Figure 2b).

ERK1/2 phosphorylation, however, was undetectable at the same time point (5 min). Thus, we performed a time course experiment to determine the best condition. As shown in Figure 2c, maximal phosphorylation occurred between 45 and 60 min after the addition of LPS to the cell cultures and was sustained for up to 120 min, starting to slowly decrease thereafter. Thus, to ensure that maximal ERK1/2 phosphorylation was achieved in all experiments, we chose the 60 min treatment period for subsequent experiments. The results obtained (Figure 2d) show that (S)-(+)-carvone was unable to decrease LPS-induced ERK1/2 phosphorylation.

### 3.2. (S)-(+)-Carvone Does Not Interfere with the Canonical NF-κB Activation Pathway

NF-κB-inducing signals, like LPS upon binding to TLR4, trigger the activation of the IκB kinase complex (IKK) which phosphorylates IκB-α, the natural inhibitor of NF-κB. Once phosphorylated, IκB-α is ubiquitinated and, subsequently, undergoes proteasomal degradation, releasing the NF-κB dimers, composed of p65 and p50 proteins, which constitutes the canonical NF-κB activation pathway. Upon release from IκB-α, the NF-κB dimers translocate to the nucleus and undergo several modifications that modulate their transcriptional activity on target genes [14].

**Figure 3:**
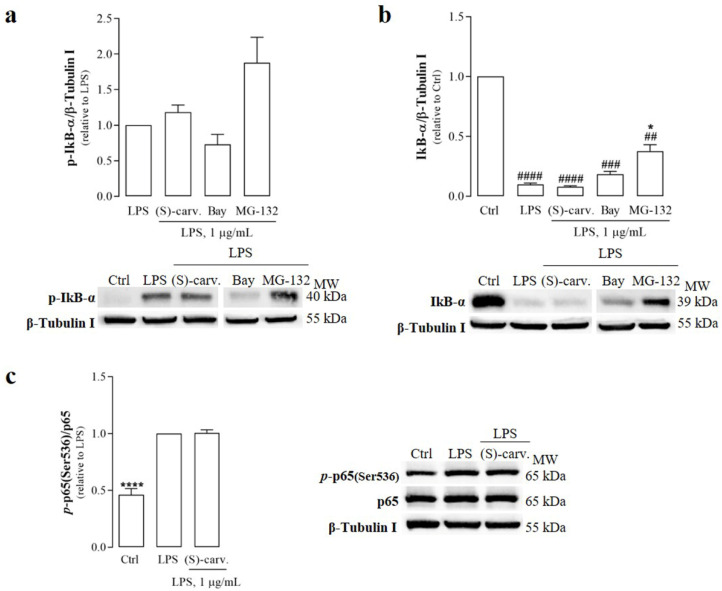
(S)-(+)-carvone does not interfere with the canonical NF-κB activation pathway, namely, phosphorylation (**a**) and degradation (**b**) of IκB-α and NF-κB/p65 phosphorylation at Ser536 (**c**) in Raw 264.7 macrophages. Macrophage cultures were treated with 1 µg/mL LPS for 5 min (**a**) or 15 min (**b**) and (**c**), following pre-treatment with the vehicle (0.1% DMSO, Ctrl), 665 µM (S)-(+)-carvone [(S)-carv.], 5 µM of the selective IKK inhibitor, Bay 11-7082, or 10 µM of the proteasome inhibitor, MG-132, for 1 h. Each column represents the mean ± SEM of five (**a**) or four (**b**) and (**c**) independent experiments. Representative images are shown. * *p* < 0.05 and **** *p* < 0.0001 relative to LPS-treated cells. ^##^ *p* < 0.01, ^###^ *p* < 0.001 and ^####^ *p* < 0.0001 relative to the Ctrl. MW: molecular weight marker.

Since the phosphorylation and degradation of IκB-α are essential for NF-κB activation, the ability of (S)-(+)-carvone to interfere with these steps was evaluated. Figure 3a,b show that LPS (1 µg/mL) induced IκB-α phosphorylation and degradation, but (S)-(+)-carvone was unable to block or even decrease those LPS-induced responses at a concentration previously observed to be sufficient to significantly decrease inflammatory gene expression [7]. On the contrary, Bay 11-7082 (5 µM), a selective NF-κB inhibitor, decreased LPS-induced IκB-α phosphorylation and degradation (Figure 3a,b), although the difference relative to cells treated with LPS alone did not reach statistical significance. Moreover, MG-132 (10 µM), a synthetic proteasome inhibitor peptide, increased the levels of phosphorylated IκB-α induced by LPS (Figure 3a), showing that its proteasomal degradation was inhibited relative to LPS-treated cells (Figure 3b). These results confirm that LPS effectively activated the canonical NF-κB activation pathway, inducing IκB-α phosphorylation and degradation, which were not affected by (S)-(+)-carvone (Figure 3a,b).

Another target of IKK is NF-κB/p65, which is phosphorylated by this kinase on the Ser536 residue located in its transactivation domain [15,16]. Therefore, and to further confirm the results obtained for IκB-α, the ability of (S)-(+)-carvone to interfere with LPS-induced NF-κB/p65 phosphorylation at Ser536 was explored. The results in Figure 3c show that the test compound was unable to decrease LPS-induced NF-κB/p65 phosphorylation on Ser536, further supporting that it does not interfere with the canonical NF-κB activation pathway.

### 3.3. NF-κB/p65 Nuclear Translocation Is Not Affected by (S)-(+)-Carvone

**Figure 4:**
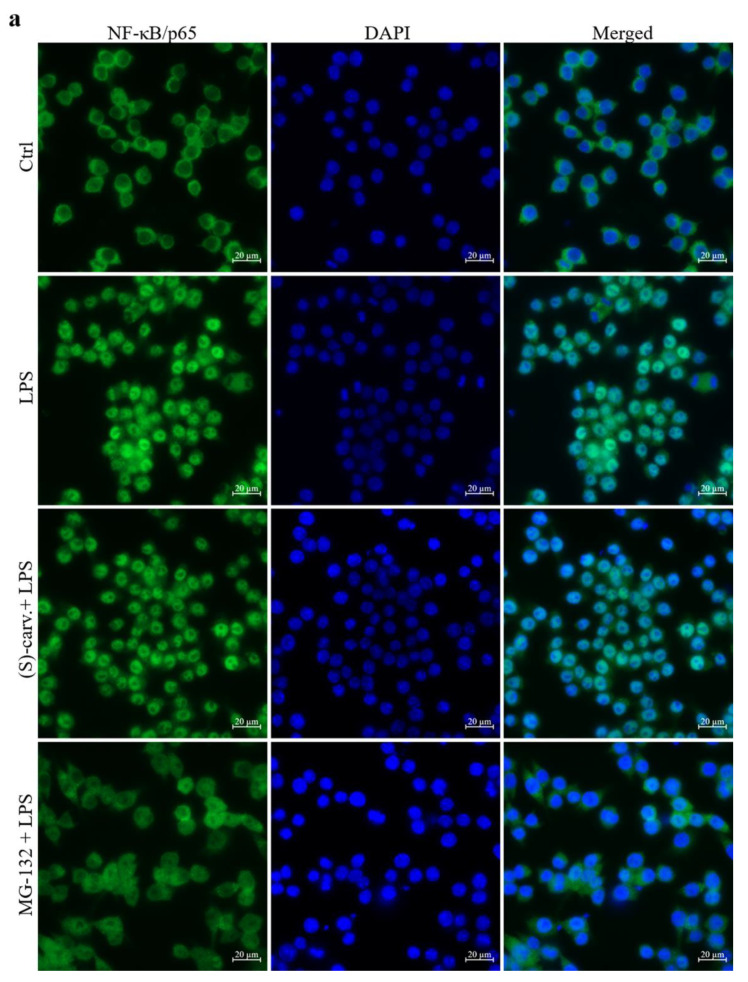

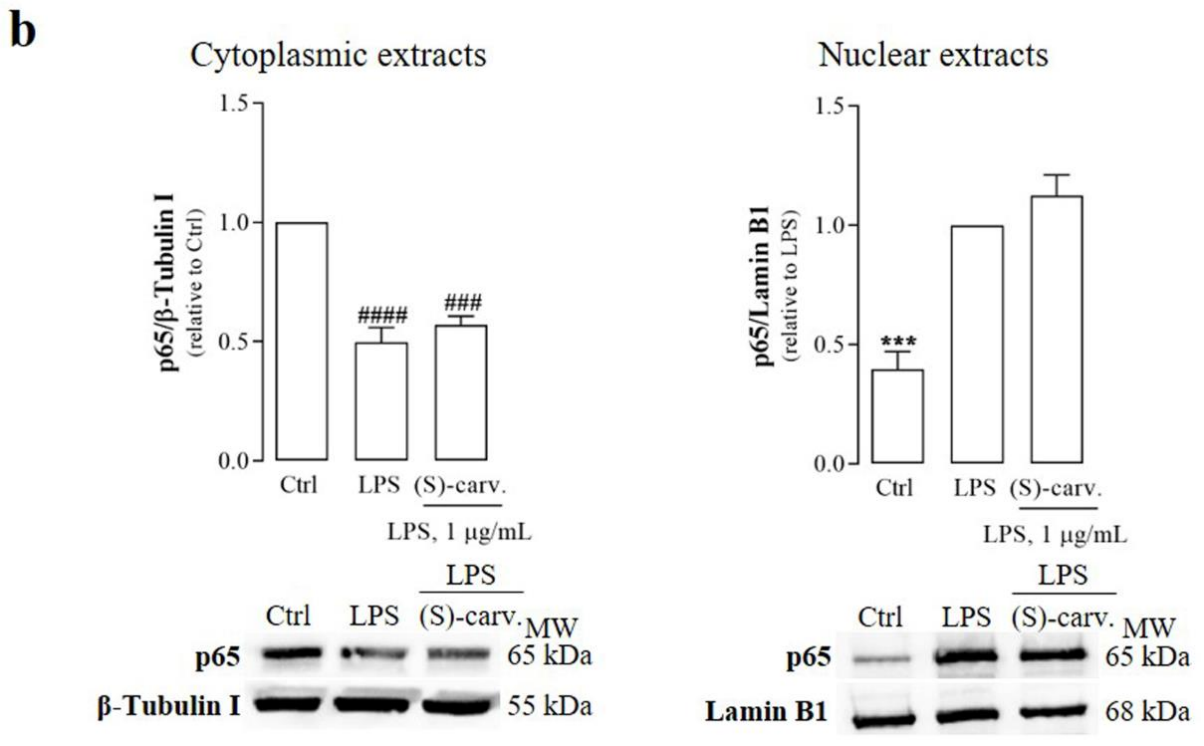
(S)-(+)-carvone does not inhibit NF-κB nuclear translocation. (**a**) Raw 264.7 macrophages were treated with 1 µg/mL LPS for 20 min, following pre-treatment with the vehicle (0.1% DMSO), 665 µM (S)-(+)-carvone [(S)-carv.] or 10 µM MG-132, for 1 h. Control cells (Ctrl) were treated with the vehicle alone. Immunofluorescence staining of NF-κB/p65 (green) and fluorescence staining of the nuclei (blue) were performed as described in Materials and Methods. Scale bar 20 µm. Representative images of each condition are shown. (**b**) Macrophages were treated with 1 µg/mL LPS for 1 h, following pre-treatment with the vehicle (0.1% DMSO) or 665 µM (S)-(+)-carvone [(S)-carv.] for 1 h. Cytoplasmic (left side) and nuclear (right side) levels of RelA/p65 were evaluated by Western blot. Control cells (Ctrl) were treated with the vehicle alone. Each column represents the mean ± SEM of three (cytoplasmic levels) and six (nuclear levels) independent experiments. Representative images are shown. *** *p* < 0.001 relative to LPS-treated cells. ^###^ *p* < 0.001 and ^####^ *p* < 0.0001 relative to the Ctrl. MW: molecular weight marker.

Since (S)-(+)-carvone was unable to prevent IκB-α phosphorylation and degradation, we hypothesized that this compound could be interfering with NF-κB nuclear translocation. To explore this possibility, immunocytochemistry was performed to detect NF-κB/p65 translocation to the nucleus. Figure 4a shows that in vehicle-treated cells (Ctrl), NF-κB/p65 immunoreactivity is clearly visible in the cytoplasm, while upon treatment with LPS (1 µg/mL), immunoreactivity is mainly located in the nucleus. Pre-treatment with 10 µM of the proteasome inhibitor, MG-132, fully inhibited NF-κB/p65 nuclear translocation, as immunoreactivity is mainly visible in the cytoplasm. Contrastingly, (S)-(+)-carvone was unable to prevent LPS-induced NF-κB/p65 nuclear translocation, as immunoreactivity is localized in the nucleus with no differences relative to cells treated with LPS alone (Figure 4a).

Confirming these results, Western blot analysis shows that treatment with LPS decreased the cytoplasmic levels of NF-κB/p65 (Figure 4b, left side), while its nuclear levels were concomitantly increased (Figure 4b, right side), but they were not affected by treatment with (S)-(+)-carvone. Thus, these results corroborate those found in the immunofluorescence assay, collectively showing that (S)-(+)-carvone does not interfere with LPS-induced NF-κB/p65 release from complexes with IκB-α and nuclear translocation.

### 3.4. NF-κB Transcriptional Activity Is Inhibited by (S)-(+)-Carvone

**Figure 5:**
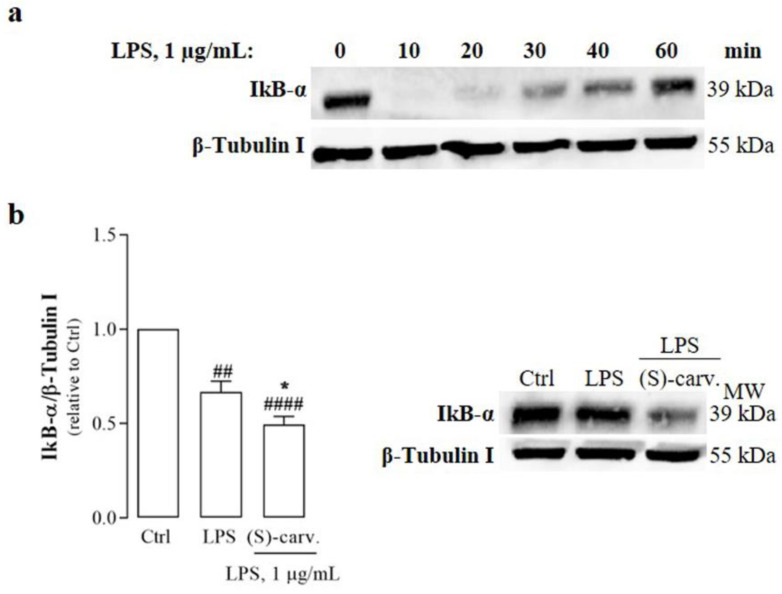
Effect of (S)-(+)-carvone on LPS-induced IκB-α resynthesis in Raw 264.7 macrophages. (**a**) Time course of LPS-induced IkB-α degradation and resynthesis. The cells were treated with 1 µg/mL LPS for the time periods indicated. (**b**) The cells were treated with the vehicle (0.1% DMSO) or 665 µM (S)-(+)-carvone [(S)-carv.] for 1 h, followed by stimulation with 1 µg/mL LPS for 1 h. Control cells (Ctrl) were treated with the vehicle alone. Each column represents the mean ± SEM of seven independent experiments. Representative images are shown. * *p* < 0.05 relative to LPS-treated cells. ^##^ *p* < 0.01 and ^####^ *p* < 0.0001 relative to the Ctrl. MW: molecular weight marker.

Although (S)-(+)-carvone did not inhibit the canonical NF-κB activation pathway, our previous work demonstrated that this compound is capable of decreasing the expression of two NF-κB target genes and major inflammatory mediators, NOS2 and IL-1β [7]. Thus, and to further elucidate these findings, the protein product of another NF-κB target gene, the IκB-α gene [17], was also evaluated. For this, we performed a time course of stimulation with LPS to determine the time points where IκB-α degradation ended and its resynthesis started. IκB-α degradation was complete within 10 to 15 min after addition of LPS, whereas its resynthesis started at 20 min and reached its maximal level within 60 min (Figure 5a). Thus, using this time point, we determined that (S)-(+)-carvone is effective in preventing LPS-induced IκB-α resynthesis (Figure 5b), supporting the hypothesis that the anti-inflammatory effects of this compound involve the modulation of NF-κB activity.

### 3.5. (S)-(+)-Carvone Promotes NF-κB/p65 Deacetylation at Lys310

**Figure 6:**
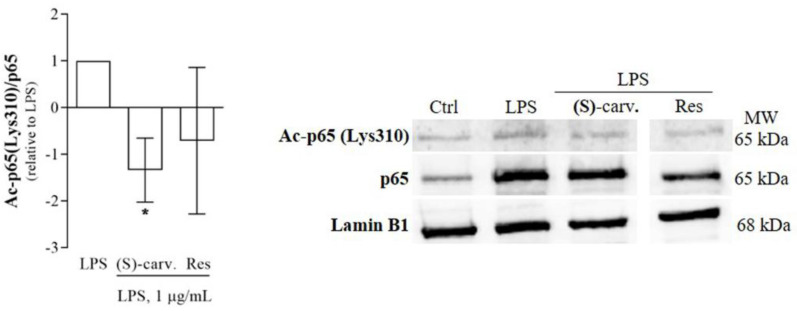
Effect of (S)-(+)-carvone on Lys310-acetylated NF-κB/p65 levels. Raw 264.7 macrophages were pre-treated with the vehicle (0.1% DMSO), 665 µM (S)-(+)-carvone [(S)-carv.] or 5.5 µM Resveratrol (Res) for 1 h, before treatment with 1 µg/mL LPS, for 1 h. Each column represents the mean ± SEM of the ratio between Ac-p65 (Lys310) and total NF-κB/p65 levels, after subtraction of the volume of the corresponding bands obtained in control cells. The results were then normalized to the ratio obtained in LPS-treated cells. The images shown are representative of four independent experiments. * p < 0.05 relative to LPS-treated cells. MW: molecular weight marker.

Besides release from IκB-α and nuclear translocation, NF-κB full transcriptional activity requires several modifications of NF-κB/p65 which impact on DNA binding affinity, interaction with coactivators and corepressors and termination of the NF-κB response [18]. Thus, we hypothesized that (S)-(+)-carvone may interfere with one or more of those modifications. Among those, NF-κB/p65 acetylation, particularly at Lys310, has been reported as essential for full NF-κB transcriptional activity [18]. Thus, we evaluated the levels of NF-κB/p65 acetylated on Lys 310 induced by LPS, in the presence and absence of (S)-(+)-carvone or resveratrol (Res), a natural polyphenolic compound known to promote NF-κB/p65 deacetylation [12] and used here as a positive pharmacological control. Figure 6 shows that treatment with LPS induced NF-κB/p65 acetylation on Lys310. Pre-treatment with Res slightly decreased the levels of acetylated Lys310 on NF-κB/p65 without reaching statistical significance, likely because Res also inhibits the canonical NF-κB activation pathway, decreasing the nuclear levels of total NF-κB/p65 [19,20], which causes the size of the effect of Res to be very small and prone to large variability. On the contrary, (S)-(+)-carvone significantly decreased those levels, suggesting that this effect can be the mechanism by which the compound inhibits NF-κB activity.

### 3.6. (S)-(+)-Carvone Activates SIRT1 without Affecting Its Protein Levels

**Figure 7:**
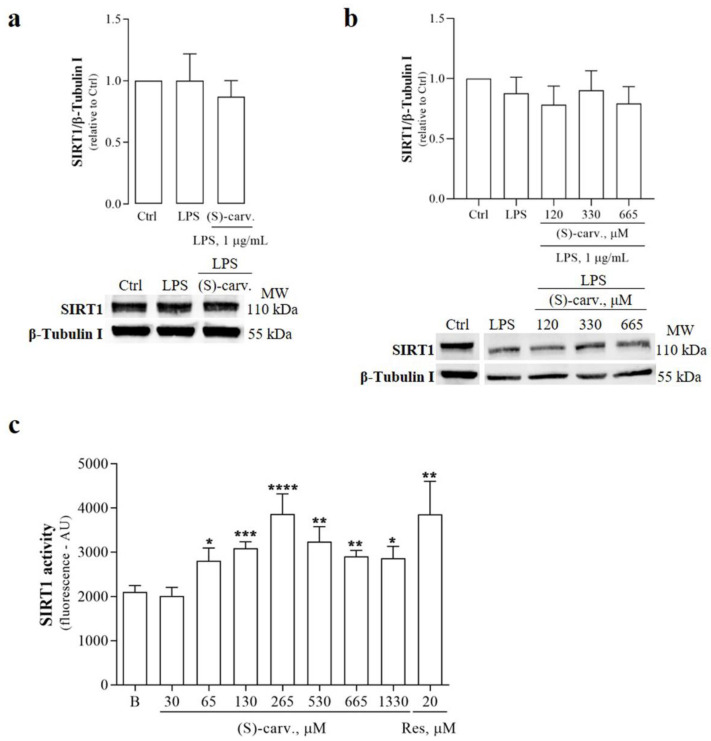
Effect of (S)-(+)-carvone on SIRT1 protein levels and activity. (**a**) and (**b**) Raw 264.7 cells were treated with 1 µg/mL LPS for 1 h (**a**) or 18 h (**b**), following pre-treatment with the vehicle (0.1% DMSO), 665 µM (**a**) or the indicated concentrations (**b**) of (S)-(+)-carvone [(S)-carv.] for 1 h. Control cells (Ctrl) were treated with the vehicle (0.1% DMSO) alone. Each column represents the mean ± SEM of three (**a**) and six (**b**) independent experiments. Representative images are shown. MW: molecular weight marker. (**c**) The activity of human recombinant SIRT1 was measured as the amount of fluorescent product released by deacetylation of a specific fluorogenic peptide substrate, in the presence or absence (basal activity, B) of the indicated concentrations of (S)-(+)-carvone [(S)-carv.] or Resveratrol (Res), used as a pharmacological control of SIRT1 activation. Fluorescence intensity is directly proportional to SIRT1 activity. Results are presented as mean fluorescence intensity in arbitrary units ± SEM. Each concentration of (S)-(+)-carvone [(S)-carv.] and Res was tested at least 6 times. * *p* < 0.05, ** *p* < 0.01, *** *p* < 0.001 and **** *p* < 0.0001 relative to the basal SIRT1 activity. B: basal SIRT1 activity.

Deacetylation of Lys310 on NF-κB/p65 is specifically mediated by Sirtuin-1 (SIRT1), a NAD^+^-dependent class III histone/protein deacetylase, leading to inhibition of NF-κB transcriptional activity [12]. Increased deacetylation of SIRT1 target proteins can occur in response to stimuli that increase the protein levels of the enzyme, with or without affecting its activity [21,22]. Thus, to further elucidate the mechanism underlying the ability of (S)-(+)-carvone to deacetylate NF-κB/p65, we evaluated the protein levels of SIRT1 upon treatment of macrophage cultures with LPS for 1 h, in the presence and absence of the test compound. Figure 7a shows that SIRT1 protein levels remained constant upon LPS treatment either in the presence or absence of (S)-(+)-carvone.

To further determine whether (S)-(+)-carvone can modulate SIRT1 protein levels independently of its effects on NF-κB activity, we tested a time point sufficiently distal to be independent of the effects on NF-κB. Figure 7b shows that even after treatment for 18 h, SIRT1 protein levels remained identical in cells treated with LPS alone and in the presence of (S)-(+)-carvone.

Then, we investigated the ability of (S)-(+)-carvone to directly enhance the activity of SIRT1. For this, we used an in vitro fluorometric assay based on the ability of recombinant human SIRT1 to deacetylate a synthetic peptide derived from p53, a prototypical SIRT1 substrate [23]. Figure 7c shows that the basal enzyme activity increased in the presence of different concentrations of (S)-(+)-carvone, reaching a maximum increase of 84% at a concentration of 265 µM. As expected, Res, a known SIRT1 activator, was also effective in increasing basal SIRT1 activity.

Taken together, the results show that (S)-(+)-carvone promoted NF-κB/p65 deacetylation, likely by directly activating SIRT1, and this mechanism probably underlies the inhibitory effect of (S)-(+)-carvone on NF-κB-dependent gene transcription.

## 4. Discussion

Inhibition of MAPK and/or NF-κB signaling pathways are mechanisms relevant to dampen chronic low-grade inflammation that are targeted by many compounds of natural origin [24,25], from polyphenols, such as Res [26], to monoterpenes, such as myrcene, limonene [27] and α-pinene [28]. Thus, to elucidate the molecular mechanism of the anti-inflammatory effects of (S)-(+)-carvone that we observed in murine macrophages and human chondrocytes [7], we started by evaluating its ability to inhibit those signaling pathways. Interestingly, the results demonstrate that only JNK1 is significantly inhibited by (S)-(+)-carvone (Figure 2b), while it does not prevent LPS-induced activation of any of the other MAPK family members to a significant extent (Figure 2a,d). Nonetheless, inhibition of JNK1 may contribute to the anti-inflammatory effects that we and others previously observed with (S)-(+)-carvone. In fact, activation of this MAPK enhances the activity of pro-inflammatory transcription factors, namely, NF-κB [29] and Activator Protein-1 [30,31], which are crucial to the expression of inflammatory mediators, such as NOS2 [32,33] and matrix metalloproteases [34,35], in response to various stimuli, in different cells.

On the other hand, (S)-(+)-carvone did not prevent any of the steps involved in the canonical NF-κB activation pathway, that is, IκB-α phosphorylation and degradation and NF-κB/p65 nuclear translocation and phosphorylation on Ser536 (Figure 3 and Figure 4). Nonetheless, we further confirmed that this compound is effective in inhibiting the expression of IκB-α, another NF-κB target gene (Figure 5).

Since several post-translational modifications of NF-κB/p65 play a critical role in modulating its DNA-binding affinity and transcriptional activity [18], we hypothesized that (S)-(+)-carvone may exert its anti-inflammatory effects by modulating such modifications. Among those post-translational modifications, acetylation of Lys310 by the co-activator and histone/protein acetyltransferase, CBP/p300, is required for the full transcriptional activity of NF-κB, without interfering with DNA binding [18]. Deacetylation of that Lys residue prevents and contributes to cease NF-κB transcriptional activity because it allows for the subsequent ubiquitination and degradation of promoter-associated NF-κB/p65 [18]. As shown in Figure 6, (S)-(+)-carvone significantly decreased the levels of NF-κB/p65 acetylated on Lys310 induced by LPS in mouse macrophages, suggesting that this can be the mechanism underlying the inhibition of NF-κB-dependent gene expression.

Decreased levels of acetylated NF-κB/p65 can occur due to the inhibition of acetyltransferase (HAT) enzymes or the activation of histone/protein deacetylases (HDAC). Among HDACs, SIRT1 has a major role in modulating NF-κB transcriptional activity by directly interacting with and deacetylating NF-κB/p65 on Lys310 [12]. Thus, we hypothesized that (S)-(+)-carvone could target SIRT1. The results presented in Figure 7 confirmed this hypothesis, showing that (S)-(+)-carvone significantly increases the basal activity of human recombinant SIRT1 without affecting its protein levels.

To our knowledge, this is the first study identifying a monoterpene compound as a direct activator of SIRT1. Although studies using (S)-(+)-carvone are scarce, previous studies using the racemic mixture containing both carvone enantiomers, (S)-(+)- and (R)-(-)-carvone, reported some pharmacological activities, including antioxidant [9], anti-inflammatory [7,8], anti-carcinogenic [36], anti-hyperglycemic and anti-hyperlipidemic [10,11] properties. Interestingly, SIRT1 activation has been shown to have a role in all these processes [37,38], suggesting that, at least in part, this can be the mechanism underlying the pharmacological activities previously reported for carvone.

Remarkably, NF-κB and SIRT1 are involved in an antagonistic crosstalk whereby SIRT1 inhibits NF-κB activity by deacetylating NF-κB/p65 while this transcription factor inhibits SIRT1 expression [39]. Decreased SIRT1 expression and increased NF-κB activity are found in many metabolic and age-related diseases, so that SIRT1 activation and NF-κB inhibition are envisaged as promising therapeutic strategies for those diseases, as well as to delay the consequences of aging [38,40,41]. Interestingly, recent studies showed that inhibitors of the sodium-glucose cotransporter-2, used in the therapy of type 2 diabetes, have cardioprotective effects in heart failure related to the systemic activation of SIRT1 and probably to the induction of SIRT1 expression in the heart [42]. Therefore, the ability of (S)-(+)-carvone to directly increase the activity of SIRT1 has huge therapeutic potential. Additional studies addressed at pharmacokinetic and further pharmacodynamic elucidation, namely, in terms of selectivity, efficacy and safety, in cell and animal models of disease are required to fully ascertain the therapeutic potential of (S)-(+)-carvone. On the other hand, the identification of a non-polyphenolic compound, (S)-(+)-carvone, as a SIRT1 activator opens up the possibility that other monoterpene compounds, in particular those that share structural features, like the *p*-menthane backbone, may also present the same property, presenting new perspectives and opportunities for the pharmacological modulation of SIRT1.

## 5. Conclusions

In summary, the results presented show that (S)-(+)-carvone, a *p*-menthane-derived monoterpene, is able to directly activate SIRT1, enhancing NF-κB/p65 deacetylation and decreasing its transcriptional activity and the consequent inflammatory response. (S)-(+)-carvone is the first non-polyphenolic compound found to directly activate SIRT1, opening up new perspectives and opportunities for the development of novel drugs to target numerous diseases in which SIRT1 plays a protective role.

## Figures and Tables

**Table 1:** List of primary antibodies used in Western blot assays.

Protein	Source	Clonality	Dilution	Supplier	Catalogue/Lot Number
IκB-α	rabbit	polyclonal	1:1000	Cell Signaling Technology, Inc., Danvers, MA, USA	#9242/9
phospho-p44/42 MAPK (ERK1/2) (Thr202/Tyr204)	rabbit	polyclonal	1:1000	Cell Signaling Technology, Inc.	#9101/27
p44/42 MAPK (ERK1/2)	rabbit	polyclonal	1:1000	Cell Signaling Technology, Inc.	#9102/26
phospho-p38 MAPK (Thr180/Tyr182)	rabbit	polyclonal	1:1000	Cell Signaling Technology, Inc.	#9211/21
p38 MAPK	rabbit	polyclonal	1:1000	Cell Signaling Technology, Inc.	#9212/17
SAPK/JNK	rabbit	polyclonal	1:1000	Cell Signaling Technology, Inc.	#9252/17
acetyl-NF-κB p65 (Lys310)	rabbit	polyclonal	1:750	Cell Signaling Technology, Inc.	#3045/2
Sirtuin-1	rabbit	polyclonal	1:1000	Sigma-Aldrich Co.	07-131/2736563
Lamin B1	rabbit	polyclonal	1:1000	Abcam, Cambridge, UK	ab16048/GR48958-1
phospho-SAPK/JNK (Thr183/Tyr185)	rabbit	monoclonal	1:1000	Cell Signaling Technology, Inc.	#4668/11
NF-κB p65 (D14E12) XP^®^	rabbit	monoclonal	1:1000	Cell Signaling Technology, Inc.	#8242/4
phospho- NF-κB p65 (Ser536)	rabbit	monoclonal	1:1000	Cell Signaling Technology, Inc.	#3033/14
phospho-IκB-α (Ser32/36)	mouse	monoclonal	1:1000	Cell Signaling Technology, Inc.	#9246/14
β-Tubulin I	mouse	monoclonal	1:20,000	Sigma-Aldrich Co.	T7816/052M4835

## Data Availability

All data are available from the corresponding author upon request.
